# Identification of basepairs within Tn5 termini that are critical sfor H-NS binding to the transpososome and regulation of Tn5 transposition

**DOI:** 10.1186/1759-8753-3-7

**Published:** 2012-04-13

**Authors:** Crystal R Whitfield, Brian H Shilton, David B Haniford

**Affiliations:** 1 Department of Biochemistry, University of Western Ontario, London, Ontario N6A 5C1, Canada

**Keywords:** Tn5, H-NS, DNA transposition, Transpososome assembly, Host factor

## Abstract

**Background:**

The H-NS protein is a global regulator of gene expression in bacteria and can also bind transposition complexes (transpososomes). In Tn5 transposition H-NS promotes transpososome assembly *in vitro *and disruption of the *hns *gene causes a modest decrease in Tn5 transposition (three- to five-fold). This is consistent with H-NS acting as a positive regulator of Tn5 transposition. Molecular determinants for H-NS binding to the Tn5 transpososome have not been determined, nor has the strength of the interaction been established. There is also uncertainty as to whether H-NS regulates Tn5 transposition *in vivo *through an interaction with the transposition machinery as disruption of the *hns *gene has pleiotropic effects on *Escherichia coli*, the organism used in this study.

**Results:**

In the current work we have further examined determinants for H-NS binding to the Tn5 transpososome through both mutational studies on Tn5 termini (or 'transposon ends') and protein-protein cross-linking analysis. We identify mutations in two different segments of the transposon ends that abrogate H-NS binding and characterize the affinity of H-NS for wild type transposon ends in the context of the transpososome. We also show that H-NS forms cross-links with the Tn5 transposase protein specifically in the transpososome, an observation consistent with the two proteins occupying overlapping binding sites in the transposon ends. Finally, we make use of the end mutations to test the idea that H-NS exerts its impact on Tn5 transposition *in vivo *by binding directly to the transpososome. Consistent with this possibility, we show that two different end mutations reduce the sensitivity of the Tn5 system to H-NS regulation.

**Conclusions:**

H-NS typically regulates cellular functions through its potent transcriptional repressor function. Work presented here provides support for an alternative mechanism of H-NS-based regulation, and adds to our understanding of how bacterial transposition can be regulated.

## Background

Most bacteria harbor a variety of different types of transposons [1]. While transposons can compromise genome stability through the various types of DNA rearrangements they promote, they can also confer a selective advantage to their hosts. This can come about through transposons acquiring genes that encode resistance to antibiotics and other environmental toxins, or through transposon insertion events that alter the expression of key host genes. In order for transposons and their hosts to coexist, transposition levels must be tightly regulated [2]. There are several examples where host proteins have been co-opted to down-regulate transposition. For instance, Dam methylase of *E. coli *methylates GATC sequences found in both the promoters controlling the expression of some transposase genes and in the transposon ends of several transposons. The former inhibits transposase expression and the latter inhibits transposase binding to transposon end sequences [3,4]. Other examples of host proteins that limit transposition include proteins that are global regulators of gene expression in bacteria, including IHF [5], RNaseE [6] and Hfq [7]. It is also apparent in some cases that there has been strong selective pressure for transposons to contain regulatory sequences for transposase genes that are suboptimal for transposase expression. For example, both Tn10 and Tn5 have weak promoter sequences and suboptimal translation initiation regions for their transposase genes. On the other hand there are some examples where transposons appear to have co-opted host proteins to promote their transposition. Examples of such proteins include IHF, HU, H-NS, Fis, topoisomerase I, DNA gyrase and DnaA [2,8].

It is often unclear as to whether host proteins directly or indirectly regulate transposition reactions. The development of *in vitro *transposition reactions for systems such as Mu, Tn7, Tn10 and Tn5 has allowed host factors implicated as regulators of transposition reactions to be tested for their potential to directly interact with the transposition machinery. IHF, H-NS and HU are all DNA-binding proteins that have been shown to directly interact with transposition complexes *in vitro *[2,8,9]. The distinction between a direct versus an indirect regulatory pathway could be important with regard to how efficiently and quickly a transposon can respond to changing physiological conditions in the cell.

In the current work we focused on the role of H-NS in Tn5 transposition. H-NS is a highly expressed DNA-binding protein that is present in many proteobacteria [10]. H-NS binding to high affinity sites embedded within A-T rich sequences is thought to nucleate polymerization of H-NS on DNA [11]. In solution, H-NS exists predominantly as a dimer at physiological concentrations [12], but upon binding DNA H-NS forms higher order oligomers through head-to-head and tail-to-tail interactions between adjacent dimers [13]. Oligomerization of H-NS on promoter regions of genes results in gene silencing probably through exclusion of RNA polymerase [14]. H-NS influences the expression of a large number of genes in *E. coli *and is therefore considered a global regulator of gene expression [15,16]. H-NS also plays an important role in lateral gene transfer in some proteobacteria as it has a propensity to silence newly acquired genes, which tend to be A-T rich, permitting bacteria to gradually integrate the new DNA into existing regulatory circuits [14].

**Figure 1:**
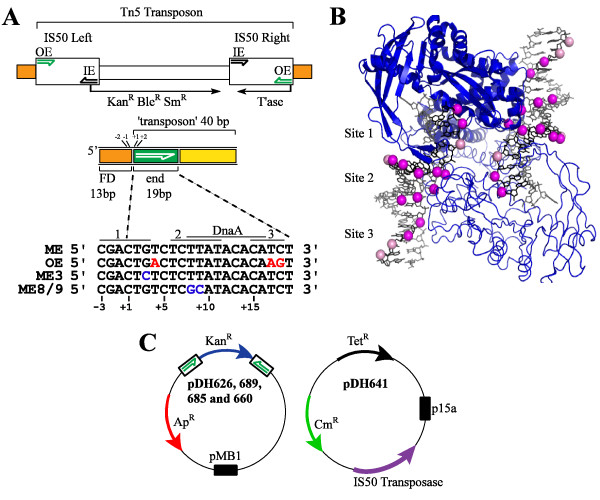
**Tn5, transpososome structure and transposon substrates**. (**A**) The organization of Tn5 (5.8 kb) is shown. The outside (OE) and inside ends (IE) of Tn5 (half arrows) contain determinants for transposase binding. IS50 Right encodes transposase and other ORF's are shown (thin arrows show the corresponding transcription unit). Flanking donor DNA is represented as orange rectangles. Below the schematic of Tn5 an illustration of the basic transposon end fragment (53 basepair) used for *in vitro *binding studies is shown. In addition, the sequence of the terminal 19 nucleotides, as well as 3 flanking nucleotides, of the non-transferred strand of each of the four transposon end substrates used in this work is shown (ME - mosaic end). Basepair changes between the ME and mutant forms of the ME (ME 3 and ME 8/9) are in blue. Basepair differences between OE and ME sequences are in red. The DnaA binding site is also shown. Potential H-NS binding sites inferred from hydroxyl radical footprinting are also shown and identified as either sites 1, 2 or 3. (**B**) Sites 1, 2 and 3 represent potential H-NS binding sites of the H-NS-Tn5 transpososome as described in (A); the light and dark pink spheres placed in the structural model of the Tn5 transpososome represent positions of weak and strong protection against hydroxyl radical attack, respectively. (**C**) Schematic of plasmids used for mating out assays. The plasmid on the left contains the mini-Tn5-Kan element with arrows in boxes depicting the end sequence (ME - pDH626, OE - pDH689, ME 3 - pDH685, or ME 8/9 - pDH660) and the thick blue arrow indicates the kanamycin resistance gene. The plasmid on the right encodes transposase (MA56) (purple arrow) under control of its native promoter. Black boxes represent the origins of replication, pMB1 and p15A; Ap^R^, Cm^R ^and Tet^R ^encode resistance genes for ampicillin, chloramphenicol and tetracycline, respectively. Note that the non-mutated ME is referred to as the wild type (WT) ME in this work.

Tn5 is a composite transposon made up of three antibiotic resistance genes encompassed by insertion sequences IS50-Right and IS50-Left (Figure 1A). Tn5/IS50 is widely distributed in proteobacteria. Tn5 and IS50 transposition is tightly regulated with events occurring at a frequency of roughly one event per element per generation in 10^5 ^and 10^3 ^cells, respectively. Transposition occurs predominantly through a cut-and-paste mechanism involving the formation of transposon end hairpins [17]. A high-resolution structure of the Tn5 transpososome has provided a wealth of information regarding protein-protein and protein-DNA interactions within the transpososome [18], but details still remain to be elucidated with regard to how the transpososome is assembled.

In previous work we have shown that inclusion of H-NS in Tn5 transpososome assembly reactions resulted in incorporation of H-NS into the transpososome. Moreover, when such assembly reactions were performed under conditions where transpososome assembly was suboptimal (that is, in the presence of a DNA competitor) it was found that inclusion of H-NS greatly facilitated transpososome formation. Importantly, H-NS did not directly impact the efficiency of transposon excision when it was added to reactions containing pre-assembled transpososomes under standard conditions [9]. The positive effect of H-NS on transpososome formation could result from H-NS promoting: (1) the formation of a pre-transpososome complex (for example, a complex where a monomer of transposase binds a single transposon end); (2) the assembly of pre-transpososome complexes into transpososome; and/or (3) the stabilization of the transpososome. Consistent with H-NS acting as a positive regulator of Tn5 transposition it has previously been shown that Tn5 transposition is reduced approximately three- to five-fold in *E. coli *containing a disruption of the *hns *gene (Δ*hns*) [9].

Our finding that H-NS binds to the Tn5 transpososome *in vitro *led us to perform DNA footprinting experiments in an attempt to localize the site(s) of binding. The reactivity of the transpososome DNA to hydroxyl radical cleavage was altered at three sites, which we have designated sites 1, 2 and 3 [9] (Figure 1). Based on the transpososome crystal structure, sites 2 and 3 are the most obviously accessible sites for H-NS binding.

In the current study we have further investigated the interaction between H-NS and the Tn5 transpososome by measuring the affinity of H-NS for the Tn5 transpososome and using site-directed mutagenesis and protein-protein cross-linking studies to define determinants for H-NS binding to the transpososome. We have also used the information gained from site-directed mutagenesis to test the idea that H-NS regulates Tn5 transposition *in vivo *by acting directly on the transpososome.

## Results

### Basepair mutations within two putative H-NS binding sites reduce the affinity of H-NS for the Tn5 transpososome

We previously used hydroxyl radical footprinting to characterize the H-NS interaction with a Tn5 transpososome assembled with mosaic end (ME) sequences [9]; the ME is a chimeric end composed of nucleotides from both the outside (OE) and inside ends (IE) of Tn5 and is optimized for use *in vitro *[19]. Three potential H-NS binding sites were defined (see model in Figure 1B). In this work we have tested the importance of each of the three sites for H-NS binding by introducing one or more basepair mutations into these sites. As H-NS binding to ME sequences is dependent on the presence of transposase [9], we targeted residues expected not to be critical for transposase-end interactions. This greatly limited the number of mutations we tested as transposase makes extensive contacts with the transposon end DNA [18]. The three different transposon end substrates we tested for H-NS binding are shown in Figure 1A.

**Figure 2:**
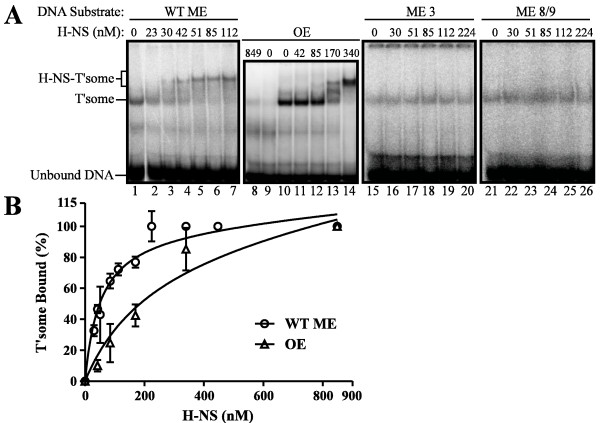
**H-NS binding assays**. (**A**) H-NS and transposase were added simultaneously to the indicated ^32^P-labeled transposon end substrate DNAs where indicated. Binding reactions were analyzed by gel electrophoresis on a native 5% polyacrylamide gel and subject to phosphorimager analysis. In each experiment transposase and substrate DNA concentrations were kept constant, while the concentration of H-NS was varied as indicated. Positions of transpososome (T'some), H-NS-shifted transpososome (H-NS-T'some) and unbound substrate DNA are indicated. In (A) lane 1 of the 'WT ME' gel was originally loaded in the last lane of the gel and was moved without alteration to the first lane of the gel. (**B**) Binding isotherms of fractional saturation as a function of H-NS concentration for H-NS binding to the wild-type ME and the OE transpososomes. Each binding isotherm was derived from four independent binding experiments similar to those depicted in (A). The percent transpososome complex shifted by H-NS was determined and these data were fit to a quadratic equation as described in Methods. The fits were used to provide estimates for the observed dissociation constant, obs K_d _for the wild type mosaic end (WT ME) and the outside end (OE).

We used an electrophoretic mobility shift assay (EMSA) to measure the impact of the above basepair changes on incorporation of H-NS into the Tn5 transpososome. In the EMSA's shown in Figure 2 we simultaneously mixed H-NS and transposase with ^32^P-labeled DNA in buffer lacking a divalent metal ion. These conditions favor the formation of an H-NS-bound transpososome, but no chemical steps in transposition take place. Under these conditions, H-NS binding reached saturation for the WT ME and OE transpososomes at concentrations of 112 nM (lane 6) and 340 nM (lane 14), respectively. This is manifested as an up-shift in the gel mobility of the respective transpososomes. Notably there was no appreciable binding of H-NS to OE substrate DNA that had not associated with transposase (lane 8). This is indicative of H-NS binding to the transpososome with high specificity. In contrast, under the same reaction conditions we did not detect H-NS binding to either transpososomes formed with ME 3 (lanes 16 to 20) or ME 8/9 (lanes 22 to 26) substrates. This clearly shows that the selected site 1 (ME 3) and site 2 (ME 8/9) mutations drastically impair H-NS binding to the transpososome without interfering with transpososome assembly.

We used binding data from EMSAs shown in Figure 2A and other similar experiments (see Additional file 1) to generate binding curves from which we could calculate K_d _values for the respective H-NS-transpososome interactions (Figure 2B). The K_d _values for these interactions are 51 ± 6.6 nM for WT ME transpososome and 232 ± 67.1 for the OE transpososome.

It should be noted that in the above experiments we do not know if H-NS is binding directly to the transpososome or to a pre-transpososome complex that is unstable in the mobility shift assay and/or converts rapidly and irreversibly to a transpososome. For convenience we will, throughout the rest of the paper, refer to the mobility shift results as H-NS binding to the transpososome.

Taken together, the results in this section are consistent with sites 1 and 2 being particularly important for H-NS binding to the transpososome. It remains to be seen if site 3 plays a critical role.

### Chemical cross-linking indicates that transposase and H-NS interact in the context of the transpososome

The results from our binding studies suggest that H-NS is in close proximity to transposase within the transpososome. Determining if there are direct interactions between transposase and H-NS has mechanistic implications for how H-NS promotes Tn5 transposition. Only a few proteins have been shown to directly interact with H-NS [20-22]. Intriguingly, one of these proteins is the Tn10 transposase. We used the same approach previously reported for the Tn10 system for assessing if Tn5 transposase directly interacts with H-NS [22]. Briefly, we treated transpososome assembly reactions (+/- H-NS) or purified Tn5 transposase (+/- H-NS) with the protein cross-linking reagent EDC/NHS; this reagent is a zero-length cross-linker that covalently links carboxyl and amino groups. Subsequently, EDC/NHS or mock treated transpososomes were gel-purified and proteins eluted from these gel slices were analyzed by Western blotting and mass spectrometry.

In the Western blot analysis we were looking for a product(s) that in the presence of EDC/NHS had a reduced mobility on an SDS gel relative to transposase (the larger of the two proteins - 60 kDa versus 16 kDa) and was detected by both antibodies to transposase and H-NS. Note that we used a version of Tn5 transposase protein containing the T7 *gene 10 *peptide as an N-terminal epitope tag, thus allowing us to use a commercially available monoclonal antibody for transposase detection. For H-NS detection we used a polyclonal H-NS antibody. After probing a blot with one antibody, the blot was stripped and re-probed with the other antibody.

**Figure 3:**
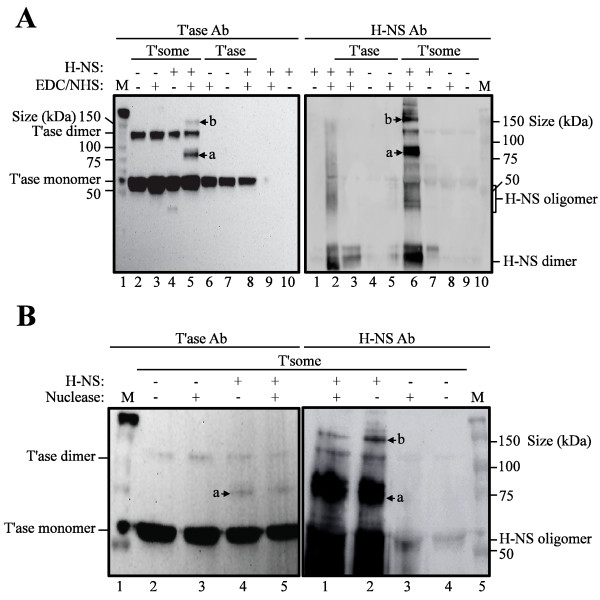
**H-NS-transposase cross-linking**. (**A**) and (**B**) Transpososomes assembled in the presence of H-NS were subjected to cross-linker (EDC/NHS) treatment as described in Methods. The resulting H-NS-transpososome was purified from a native polyacrylamide gel and proteins eluted from this gel slice were subjected to Western blot analysis using a monoclonal antibody to the T7 *gene 10 *epitope present on the N-terminus of transposase. Subsequently, the blot was stripped and re-probed with a polyclonal antibody to H-NS. Signal from the marker lane was used to align each pair of blots. In (A) EDC/NHS cross-linking was also carried out on reactions containing purified transposase and H-NS in the absence of DNA. In (B) aliquots of cross-linked H-NS-transpososome were treated with micrococcal nuclease prior to Western blot analysis. T'some, gel-purified H-NS-transpososome; T'ase, purified transposase protein; 'a' and 'b', products inferred to contain transposase cross-linked to H-NS.

**Figure 4:**
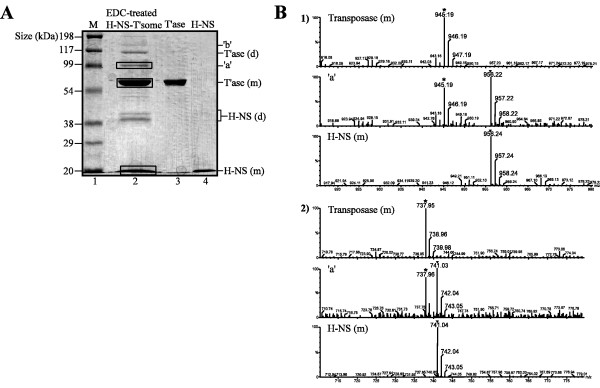
**Mass spectrometry of cross-linked product 'a'**. (**A**) Coomassie blue-stained SDS gel containing gel-purified, EDC/NHS cross-linked H-NS-transpososome (lane 2), purified transposase (lane 3) and purified H-NS (lane 4). Plugs of acrylamide from the boxed areas including transposase, H-NS and product 'a' were excised using a 'spot-picker', digested with trypsin, and analyzed by MALDI-TOF. (**B**) Two sets of spectra derived from transposase monomer (m), H-NS monomer and 'a' bands are shown. In both sets the spectra from 'a' have peptides that are also found in the spectra derived from transposase and H-NS. Peaks are separated on the x-axis based on mass/charge ratio. *, peptides corresponding to transposase;filled triangle, peptides corresponding to H-NS. **MALDI-TOF**, matrix-assisted laser desorption/ionization-time of flight.

We show in Figure 3A that EDC/NHS treatment of a transpososome assembly reaction containing H-NS yielded two prominent novel products 'a' and 'b' that were detected by both antibodies and have an apparent molecular mass greater than the mass of monomeric transposase (lane 5, left panel; lane 6, right panel). Products 'a' and 'b' were not detected in the mock cross-linking reaction where EDC/NHS was omitted (lane 4, left panel; lane 7, right panel) and when H-NS was not included in the assembly reaction (lane 3, left panel; lane 8, right panel). Products 'a' and 'b' were also not detected when transposase was mixed with H-NS in the absence of ME DNA (lane 8, left panel; lane 3, right panel), indicating that the appearance of these products is dependent on transpososome formation. However, products 'a' and 'b' were detected after micrococcal nuclease treatment of gel-purified EDC/NHS-treated H-NS-transpososomes indicating that these products do not include a DNA component (Figure 3B). Finally, gel slices containing products 'a' and 'b' were digested with trypsin and analyzed using mass spectrometry (matrix-assisted laser desorption/ionization-time of flight (MALDI-TOF)) (Figure 4). The spectra obtained were compared to a theoretical prediction of mass-to-charge ratios of the resulting peptides from a tryptic digest of transposase and H-NS. Several peaks/peptides corresponding to both proteins were detected in 'a' samples providing definitive proof that H-NS and transposase are present in a product whose formation is dependent on EDC/NHS treatment. Unfortunately, the mass spectrometry did not provide information regarding residues involved in the cross-linking as there were no peaks/peptides that were specific to the cross-linked products.

At this point we can only speculate on the precise composition of products 'a' and 'b' based on their relative abundances and apparent molecular weights. Product 'a' is the more abundant product and accordingly is most likely the simplest in terms of composition. With an apparent molecular weight greater than 60 and less than 120 kDa, we expect this product includes a monomer of transposase cross-linked to either a monomer of H-NS (76 kDa) or a cross-linked dimer of H-NS (92 kDa). Product 'b' could be a cross-linked transposase dimer that is itself cross-linked to an H-NS monomer or a cross-linked H-NS dimer. As H-NS readily forms dimers in solution that are efficiently cross-linked by EDC/NHS [12], it is tempting to speculate that each of products 'a' and 'b' includes a cross-linked H-NS dimer.

Overall the results in this section indicate that within the Tn5 transpososome H-NS is in close enough proximity to directly interact with transposase. Given that H-NS is a DNA-binding protein, this raises the possibility that H-NS may help tether transposase to transposon end sequences by interacting both with transposase and DNA.

### Modeling H-NS into the Tn5 transpososome structure

**Figure 5:**
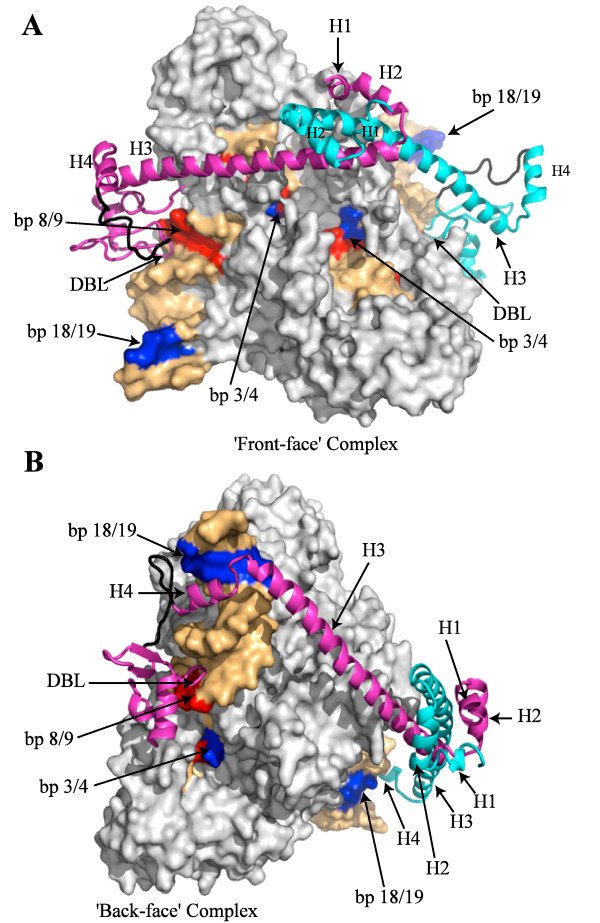
**Models for H-NS binding to the Tn5 transpososome**. (**A**) and (**B**) Models wherein a head-to-head dimer of H-NS is docked to either the 'front' or 'back' face of the transpososome. Helices 1 to 4 of H-NS are labeled, as are specific basepairs in the transposon ends that were mutated in this study (red). Individual monomers of H-NS are in different colors (cyan and magenta) and the residues linking H4 and the DNA-binding domain of H-NS are in black. Tn5 transposase residues are in grey and end sequence is in gold, blue or red. DBL; DNA-binding loop.

Our biochemical data is most consistent with a dimer of H-NS binding the Tn5 transpososome through interactions with either segment 1 or 2 of the transposon end DNA. In an attempt to integrate this data with the available structural data, we asked if an H-NS dimer could be docked into the existing Tn5 transpososome structure. An NMR structure is available for the H-NS DNA-binding domain, residues 91 to 137 [23]. On the basis of chemical shift experiments, Gordon and co-workers modeled the H-NS binding domain into a DNA duplex containing a 5'ATATAT 3' sequence. We used this model of the DNA/H-NS complex to help position the H-NS DNA-binding domain in the minor groove of the transposon end DNA in the Tn5 transpososome [18], close to positions 8 and 9; recall that mutations at these positions abrogated H-NS binding to the transpososome and these residues are within the only A-T rich stretch of the terminal portion of the ME. A second H-NS DNA-binding domain was placed in the equivalent position on the second transposon end. Positioned in this manner, the distance between the two DNA-binding domains is approximately 80 Å (Figure 5).

A crystal structure is available for residues 2 to 82 of the N-terminal oligomerization domain of H-NS [13]. This domain consists of four α-helices (H1, H2, H3 and H4) that form an extended dimer. The distance between the C-terminal ends of H3 in the H-NS dimer is approximately 100 Å, which is close to the distance of 80 Å between the H-NS DNA-binding domains that were positioned in the Tn5 transpososome (Figure 5). On this basis, we were able to connect the ends of the H-NS N-terminal domain dimer to the C-terminal DNA-binding domains using the flexible linker, residues 83 to 91 (Figure 5A). In this configuration, the H-NS dimer runs across the region of the transpososome implicated in target capture [24]. However, given the flexibility in the position of H4 and residues 83 to 91, the H-NS dimer could also cross the opposite side of the transpososome (Figure 5B). In this case, H4 was positioned to pass through the major groove of the transposon end DNA to facilitate connection with the DNA-binding domain. In this configuration H-NS could remain bound to the transpososome without interfering with target capture.

There are many possible ways the dimerization domain can be oriented relative to the DNA-binding domain and this orientation will dictate where along the transpososome face H1-H3 is positioned. At present we have chosen an orientation between the domains that limits the number of steric clashes between H1-H3 and either the 'front' or 'back' face of the transposase dimer. While the models are obviously preliminary, we think they are useful because they show that the dimensions of the head-to-head H-NS dimer and the transpososome are compatible for binding in a manner where residues established to be important in transpososome-H-NS interactions (positions 8 and 9 of the transposon ends) are the main anchor points of the structure. It should also be noted that we were unable to dock the H-NS dimer into segment 1 of the transpososome because of steric clashes with transposase.

### Mutations within H-NS binding sites 1 and 2 reduce the ability of H-NS to regulate Tn5 transposition

Identifying transposon end mutations that reduce H-NS binding to the transpososome provided us with an opportunity to test the idea that the role H-NS plays in Tn5 transposition *in vivo *is a direct result of H-NS binding to the transpososome, as opposed to being an indirect effect. The latter is a concern given the large number of genes H-NS regulates and the pleiotropic nature of *hns *mutations [10,14]. If H-NS regulates Tn5 transposition directly by acting on the transpososome, then mutations that reduce H-NS binding to the transpososome should reduce the sensitivity of transposition to the *hns *status of the cell. In other words, the roughly three- to five-fold increase in transposition of Tn5 observed in *hns + *versus Δ*hns *should be reduced when Tn5 contains a mutation in the transposon ends that reduces H-NS binding.

To test our hypothesis we generated a series of plasmids containing mini-Tn5 transposons with either WT ME's, mutant ME's or OE's. We then measured the transposition frequency of these transposons in isogenic *hns+*/Δ*hns *strains of *E. coli *using a mating out assay. In this experimental set-up transposase (M56A) was provided on a second plasmid under the control of its native promoter - the M56A mutation in transposase ensures that the transposase inhibitor protein is not synthesized making it easier to detect transposition events [25].

**Figure 6:**
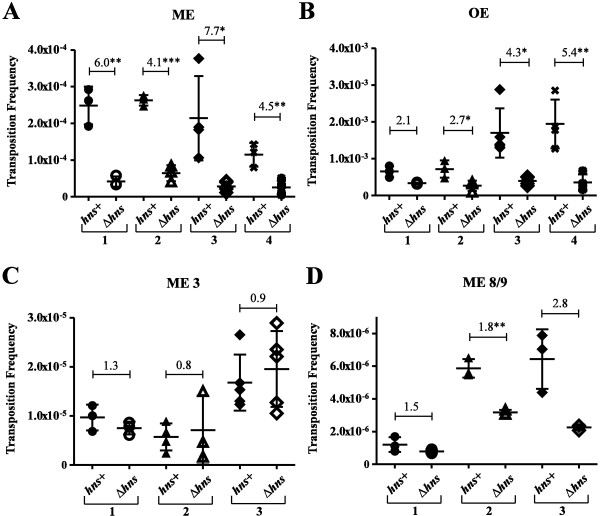
**Transposition frequencies of mini-Tn5 derivatives in isogenic *hns + *and Δ*hns *strains**. Mating out experiments were carried out on *hns + *and Δ*hns *strains of *E. coli *containing a plasmid encoding one of the mini-Tn5 derivatives indicated and a compatible plasmid encoding transposase (M56A) expressed from its native promoter (see Methods for details). Each graph provides transposition frequencies of individual clones (three to five per strain) for at least three and up to four independent experiments. Each experiment compared transposition levels for a pair of isogenic strains containing the same mini-Tn5 substrate. Horizontal and vertical lines are the mean and the standard deviation, respectively, for the transposition frequencies. The fold difference between *hns + *and Δ*hns *strains for each experiment is indicated in the upper portion of each graph. The t-test to measure variance was used to determine if differences between frequencies within each mating out experiment were/were not due to chance. *P <0.05, **P <0.01, ***P <0.001.

The results for this experiment are presented in graphical form in Figure 6 (see also Additional file 2). Each graph shows the range of transposition frequencies obtained for at least three and up to five independent donor strain clones in multiple pair-wise (*hns + *versus Δ*hns*) comparisons for a given transposon (each pair contains results from a single experiment). For each pair the 'fold difference' in transposition observed is indicated on the scatter plot. The results show a trend that generally matches the *in vitro *H-NS-transpososome binding data for the different substrates. H-NS had the highest affinity for the WT ME and there was a consistent trend of reduced transposition in Δ*hns *versus *hns + *(on average 5.6-fold) for this substrate (Figure 6A). Relative to the WT ME, the OE transpososome had the next highest affinity for H-NS and the OE transposon was slightly less sensitive relative to the WT ME transposon to *hns *status as transposition was reduced to a lesser degree (on average 3.6-fold) in Δ*hns *versus *hns + *(Figure 6B). Finally, ME 8/9 and ME 3 transpososomes had the lowest affinity for H-NS and the *hns *status of the donor strain had little impact on the transposition frequency of the corresponding transposon substrates (no effect for ME 3 and a two-fold effect for ME 8/9) (Figures 6C and 6D).

It should be noted that each of the mutant ME transposons did transpose at a lower frequency than the WT ME transposon (10-fold for ME 3 and 50-fold for ME 8/9), indicating that the mutations do have a negative impact on transposition independent of their effects on H-NS binding. As an additional control we measured relative copy number levels for the two plasmids present in each strain to ensure that the mating out results did not reflect differences in plasmid copy number. We show in Additional file 3 that plasmid copy number is not affected by *hns *status.

### H-NS status does not influence Tn5 transposase steady-state mRNA levels

**Figure 7:**
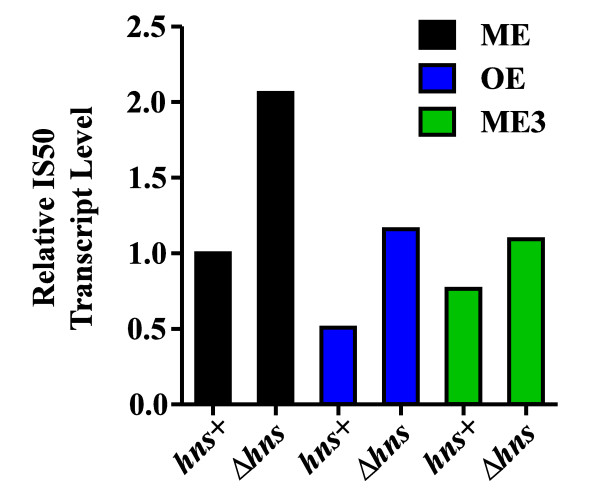
**Quantitative RT-PCR analysis of transposase mRNA levels in *hns + *and Δ*hns*, strains**. Total RNA from donor strains used in mating out experiments was isolated at mid-log phase of growth. Roughly equivalent amounts of RNA were reverse transcribed using transposase-specific primers and transposase levels were quantified using real-time RT-PCR (see Methods). We simultaneously measured 16S rRNA levels from *hns + *and Δ*hns *cells. The relative levels of IS50 transposase transcript presented were normalized to the corresponding levels of 16S rRNA transcript. Each normalized value represents an average from four independent clones from a single mating out experiment.

If H-NS stimulates Tn5 transposition by acting directly on the transpososome, we would not expect the difference in transposition frequency in *hns + *versus Δ*hns *to be linked to differential expression of the transposase gene. As H-NS is a global regulator of gene expression, it was important to rule out this possibility. We measured steady-state transposase transcript levels by quantitative RT-PCR (qRT-PCR) using RNA isolated from *hns + *and Δ*hns *cells used in mating out experiments. The results of this analysis show that transposase expression levels are marginally lower in *hns + *versus Δ*hns *cells (Figure 7; also see Additional file 4 for the raw data used to generate Figure 7). The data shown have been normalized to 16S rRNA levels measured in the two strains; 16S rRNA levels are known not to be influenced by H-NS under the conditions used in this work [26]. Thus, the higher transposition frequencies observed in *hns + *versus Δ*hns *strains cannot be accounted for by reduced transposase gene expression in Δ*hns*.

## Discussion

H-NS promotes Tn5 transpososome formation *in vitro *through an as yet undefined mechanism that involves incorporation of H-NS into the transpososome [9]. In this work we measured the binding affinity of H-NS for the Tn5 transpososome (or possibly a pre-transpososome transposition complex) and went on to identify, through mutational analysis, basepairs within Tn5 end sequences that play an important role in this interaction. We have also shown through protein-protein cross-linking analysis that H-NS directly interacts with transposase, specifically in the context of the transpososome, and presume that this interaction contributes significantly to the relatively high affinity with which H-NS binds the transpososome. Defining mutations within the Tn5 ends that strongly decreased H-NS binding to the transpososome afforded us the opportunity to ask if H-NS promotes Tn5 transposition *in vivo *by directly binding the transpososome. We found mutations that inhibited H-NS-transpososome interactions *in vitro *reduced the sensitivity of transposition reactions to the *hns *status of the cell, a finding consistent with H-NS acting directly on the transpososome to promote transposition.

### Transposon end sequences and transposase provide determinants for H-NS binding to the Tn5 transpososome

H-NS typically binds AT-rich DNA within promoter sequences independent of interactions with other proteins [14]. Within this context a wide range of binding affinities have been reported ranging from micromolar to low nanomolar. One of the tightest interactions reported to date for *E. coli *H-NS involves the promoter sequence of the *proU *operon. A 10 basepair segment within this regulatory sequence was found to bind H-NS with a K_d _of 15 nM and to serve as a nucleating sequence for H-NS binding to nearby sites possessing intrinsically lower H-NS binding affinities [11]. The highest affinity interaction for H-NS reported to date is with a Tn10 transpososome where the reported K_d _was approximately 0.3 nM. In this case, H-NS binding determinants included both transposon end sequences and the Tn10 transposase protein [22]. In the current study we measured the binding strength of H-NS for Tn5 transpososomes containing different transposon end sequences and report a K_d _value of approximately 51 nM for the WT ME end sequence. We have also shown that H-NS interacts with Tn5 transposase and accordingly infer that the transposase protein also provides determinants for H-NS binding.

H-NS could promote transpososome assembly by directly binding to the fully-assembled transpososome and stabilizing this structure. Interestingly, in the Tn10 system H-NS binds the transpososome and alters the conformation of this structure in a manner that promotes intermolecular transposition events [8,27]. Given that H-NS interacts directly with the Tn5 transposase protein, it is possible that H-NS could alter the structure of the transposase dimer and in so doing increase transpososome stability. Alternatively, H-NS could promote transposase binding to a transposon end thereby facilitating formation of a single-end complex and/or promoting the pairing of single-end complexes. As transpososomes tend to be inherently stable structures [28,29], we favor the idea that H-NS acts prior to transpososome formation. Consistent with this we have previously shown that H-NS can bind a single-end Tn5 transpososome complex [9]. Unfortunately, to this point, a Tn5 single-end complex has only been detected using a mutant form of transposase that is unable to transition into a transpososome [30], so it has not yet been possible to further dissect the role of H-NS in transpososome formation.

The DnaA protein can also bind Tn5 end sequences and could potentially compete with H-NS for binding; both the ME and OE contain a single DnaA binding site [31] and this site overlaps putative H-NS binding sites 2 and 3 (Figure 1A). Interestingly, it has been reported that DnaA can out-compete transposase for OE binding even after a transpososome has been formed [17]. This raises the possibility that *in vivo *transposase loading onto an OE or ME may require an additional host factor such as H-NS. The approximate K_d _of DnaA for a DNA sequence with a single DnaA site is 30 to 50 nM [32] and keeping in mind our determination of a K_d _of 51 nM for the H-NS interaction with Tn5 ME-transpososome, it is possible that H-NS would be able to effectively compete with DnaA for ME binding *in vivo *and thereby significantly contribute to transpososome assembly. Notably, both H-NS and DnaA are highly expressed proteins, although interestingly DnaA expression is growth rate regulated [33].

### Point mutations in putative H-NS binding sites within ME sequences greatly reduce H-NS binding to the Tn5 transpososome

The results from EMSA studies performed here are consistent with sites 1 and 2 but not site 3 harboring the most critical determinants for H-NS binding. Recent structural studies suggest a straightforward explanation for why site 2 mutations block H-NS binding to the Tn5 transpososome. NMR studies are consistent with H-NS interacting with DNA through the minor groove over five consecutive residues. In addition, results from a protein-binding microarray study revealed that the major determinant for optimal H-NS binding is the shape of the minor groove, which is dictated by local DNA sequences [23]. Mixed AT-rich sequences or A-tracts within a GC-rich sequence appear to have the optimal minor groove geometry for H-NS binding and GC basepairs in the center of an AT-tract are unfavorable for H-NS binding because of the less optimal electrostatic potential for binding arginine residues, which are present in the DNA-binding motif (an AT-hook-like loop) of H-NS [34]. Also, the presence of a 2-NH_2 _group on G that protrudes into the minor groove may provide a steric block to H-NS binding. Tn5 transposase makes extensive contacts in the major groove of the transpososome, including base-specific contacts spanning residues 7 to 13. In contrast, the minor groove is much more exposed. This includes the only A-T rich segment within the terminal 20 basepairs, which spans residues 8 to 12 [18]. As we have mutated residues 8 and 9 from T:A to G:C and C:G basepairs respectively, and seen a drastic reduction in H-NS binding, it seems likely given the preference of H-NS for AT-rich sequences imbedded in GC-rich sequences, that these mutations would directly inhibit H-NS binding. In support of this possibility we were able to dock *in silico *a head-to-head dimer of H-NS into the Tn5 transpososome using the exposed minor groove in each of the A-T rich segments of the transposon ends as anchor points for the C terminal DNA-binding domain of H-NS. It is less obvious why the mutation in putative H-NS binding site 1 would strongly inhibit H-NS binding as there does not appear to be room for H-NS to bind this segment of the transpososome.

### Genetic evidence that H-NS regulates Tn5 transposition by binding directly to transposition complexes

Identification of transposon end mutations that strongly interfere with H-NS binding to the Tn5 transpososome allowed us to test the idea that H-NS up-regulates Tn5 transposition *in vivo *by acting directly on transposition complexes. Our expectation was that the transposition frequency of a transposon harboring such a mutation(s) would not be influenced by the *hns *status of the cell. Consistent with the idea that H-NS acts directly on the transpososome to promote Tn5 transposition, we found that the transposition frequency of Tn5 elements harboring end mutations that strongly interfered with H-NS binding to the transpososome *in vitro *was largely insensitive to *hns *status. The interpretation of these results is complicated by the fact that the end mutations do more than disrupt H-NS binding to the transpososome as evidenced by the significantly lower transposition frequencies of the mutant versus wild type transposons. It is therefore possible that H-NS is unable to stimulate transposition of the mutant transposons because it cannot overcome the 'H-NS independent' defect caused by the mutations. For example, if the 'H-NS independent' defect were downstream of transpososome formation (perhaps one of the chemical steps in transposition), a boost in transpososome formation in the presence of H-NS might not have a significant rescuing effect on transposition. Alternatively, if the 'H-NS independent' defect caused by the end mutations was either upstream of transpososome formation (perhaps initial binding of transposase to an end) or at transpososome formation, and was significantly stronger than the positive effect of H-NS on transposition, then the positive effect of H-NS might be masked. We think this latter scenario is unlikely because we did not see a major negative impact of the end mutations on transpososome assembly *in vitro *in experiments presented in Figure 2. Further testing of the idea that H-NS regulates Tn5 transposition by directly interacting with the transpososome or pre-transpososome complexes will clearly require biochemical analyses of Tn5 transpososomes formed *in vivo*. However, at this point our genetic and *in vitro *biochemical analyses, as well as our qRT-PCR analysis on H-NS effects on transposase expression, provide an initial level of support for this model.

## Conclusions

As transposons have a major impact on the structure/composition of bacterial genomes and on gene expression networks, it is important to understand how their mobilization is regulated. The molecular basis of positive regulation of Tn5 transposition by H-NS is not well understood and is particularly intriguing because H-NS typically functions as a negative regulator of transcription. A better understanding of how H-NS regulates Tn5 transposition has the potential to define an atypical regulatory mechanism for H-NS. In the current work we have made progress in defining how H-NS interacts with the Tn5 transpososome. We have shown that binding of H-NS to the Tn5 transpososome *in vitro *is dependent on its interactions within the terminal nine residues of the transposon and potentially with the transposase dimer, the protein core of the transpososome. Docking studies carried out *in silico *are consistent with this interpretation. We have also provided evidence that H-NS interactions with basepairs within the terminal nine residues of Tn5 ends are important for H-NS-mediated regulation of Tn5 transposition *in vivo*.

## Availability of supporting data

The data sets supporting the results of this article are included within the article (and its additional files).

## Methods

### Plasmids and transposon DNA's

Plasmid-based mini-Tn5 elements were constructed using a three-fragment cloning strategy. Primers containing a Pst1 site (CW1 and CW2) were designed to the 5' and 3' ends of the kanamycin resistance gene from pNK1182 [35] and a PCR reaction was performed to amplify the Kan^R ^fragment. After digestion with PstI the Kan^R ^fragment was ligated to the linear fragment of KpnI-digested pTZ18U [36], and to a 20 basepair ME fragment (CW3/4 for WT ME, CW5/6 for OE, CW7/8 for ME 3 and CW9/10 for ME 8/9) containing PstI and KpnI overhangs. The resulting plasmids, pDH626, pDH689, pDH685 and pDH660, contain identical mini-Tn5 transposons except that the transposon ends were either wild-type ME, OE, ME 3 and ME 8/9 sequences, respectively. To provide a source of Tn5 transposase on a compatible plasmid to the above transposon substrates, Tn5 transposase DNA from pRZ9905 [37] was cloned into pACYC184 as follows: restriction sites for either HindIII or XbaI were incorporated into primers (CW11 and 12) complementary to the 5' and 3' ends of the Tn5 transposase gene and following amplification and digestion of the transposase fragment with HindIII and XbaI the transposase fragment was cloned into HindIII/XbaI-digested pACYC184. The transposase gene used contains a mutation that eliminates synthesis of the inhibitor protein, but otherwise is wild-type in sequence.

Transposon end substrates used for binding assays contain 13 basepairs of donor DNA and 40 basepairs of transposon DNA. These substrates were generated by annealing complementary, gel-purified oligonucleotides (Table 1) and were subsequently 5' end-labelled with T4 polynucleotide kinase (New England Biolabs - Ipswich, MA, USA) and γ^32^P-ATP (Perkin-Elmer - Boston, MA, USA) using standard procedures.

**Table 1:** Oligonucleotides used in this study^a^.

Name	Sequence (5' to 3')
CW1	CGCGTTTAATCTGCAGCACAGTCGTGATGGC

CW2	CCCTGCGCAGCGCGCAGCTGCAGCCTGAATACGCG

CW3	CTCGA**C**TGTCTCTTATACACATCTAGCGTCCTGAACGGAACCTTCTGCA

CW4	GAAGGTTCCGTTCAGGACGCTAGATGTGTATAAGAGACA**G**TCGAGGTAC

CW5	CTCGA**C**TGACTCTTATACACAAGTAGCGTCCTGAACGGAACCTTCTGCA

CW6	GAAGGTTCCGTTCAGGACGCTACTTGTGTATAAGAGTCA**G**TCGAGGTAC

CW7	CTCGA**C**TCTCTCTTATACACATCTAGCGTCCTGAACGGAACCTTCTGCA

CW8	GAAGGTTCCGTTCAGGACGCTAGATGTGTATAAGAGAGA**G**TCGAGGTAC

CW9	CTCGA**C**TGTCTCGCATACACATCTAGCGTCCTGAACGGAACCTTCTGCA

CW10	GAAGGTTCCGTTCAGGACGCTAGATGTGTATGCGAGACA**G**TCGAGGTAC

CW11	NNNAAGCTTGGGTAACGCCAGGGTTTTCCCACTC

CW12	NNNTCTAGACGCCAAGCTTGCATGCCTGCAGGTC

WT ME 53NTS	CCCTGCAGGTCGA**C**TGTCTCTTATACACATCTTGAGTGAGTGAGCATGCA

WT ME 53TS	ACATGCATGCTCACTCACTCAAGATGTGTATAAGAGACA**G**TCGACCTGCAGGG

OE 53NTS	CCCTGCAGGTCGA**C**TGACTCTTATACACAAGTTGAGTGAGTGAGCATGCA

OE 53TS	ACATGCATGCTCACTCACTCAACTTGTGTATAAGAGTCA**G**TCGACCTGCAGGG

ME 3 53NTS	CCCTGCAGGTCGA**C**TCTCTCTTATACACATCTTGAGTGAGTGAGCATGCA

ME 3 53TS	ACATGCATGCTCACTCACTCAAGATGTGTATAAGAGACA**G**TCGACCTGCAGGG

ME 8/9 53NTS	CCCTGCAGGTCGA**C**TGTCTCGCATACACATCTTGAGTGAGTGAGCATGCA

ME 8/9 53TS	ACATGCATGCTCACTCACTCAAGATGTGTATGCGAGACA**G**TCGACCTGCAGGG

T1TaseF	GACCTCTTAAGATGGTAACGTTCATG

T1TaseR	GCCGAAGAGAACACAGATTTAGC

T1TaseProbe^b^	6FAM-TAACTTCTGCTCTTCATCGTG-MGBNFQ

16SF	ACCAGGGCTACACACGTGCTA

16SR	TCTCGCGAGGTCGCTTCT

16SProbe^b^	6FAM-AATGGCGCATACAAA-MGBNFQ

^a^The first nucleotide of the transposon end sequence (1 or +1 position) of each strand is in bold. The mutated nucleotides are underlined. ^b^6FAM represents 6-carboxyfluorescein. MGBNFQ represents the quencher dye.

### Protein purification

Tn5 transposase and H-NS were purified as described previously [38,39]. Tn5 transposase concentration was determined using the Bradford assay (Pierce - Rockford, IL, USA) and H-NS concentration was determined using the BCA assay (Pierce).

### H-NS binding assay

Transpososome assembly reactions were performed by mixing ^32^P-labelled transposon end fragments (2 nM) and purified transposase (200 nM) as described previously [9], except that transposase was added at 400 nM in the case of reactions with the ME 8/9 substrate in order to obtain roughly equivalent amounts of transpososome in all *in vitro *reactions. H-NS was added to the reactions at the same time as transposase in varying concentrations (normally 23 nM to 849 nM) and after incubation for 30 minutes at 37°C reactions were mixed with load dye and applied to a 5% native polyacrylamide gel. Gels were run and analyzed as previously described [9]. The typical transpososome yield was about 10% of input DNA giving a final concentration of 0.2 nM transpososome per assembly reaction. ImageQuant v5.1 software was used to analyze H-NS-bound fractions of transpososome based on the proportion of labelled DNA present in the mobility shifts compared to the overall total labelled DNA in each lane. The equilibrium dissociation constant (K_d_) was determined using the equation: θ^-1 ^= 1 + (K_d_/[P_*t*_]) where θ is the fraction of H-NS-bound transpososome and P_*t *_is the total H-NS concentration. At least three independent binding experiments were performed for each transposon end substrate. Data for WT ME and OE substrates was fit to a quadratic equation using non-linear regression and binding curves were generated using GraphPad Prism v5.0.

### Mating out assay

Mating out assays were performed in isogenic *hns + *(DBH33) and Δ*hns *(DBH1) donor strains (Table 1) as previously described [9]. These strains were transformed with a 'transposon substrate' plasmid containing a Kan^R ^gene between the transposon ends (either pDH626, pDH689, pDH685 or pDH660) and a compatible 'transposase' plasmid (pDH641). Donor cells were mixed with recipient cells (HB101) and after growth in LB mating mixes were pelleted and resuspended in 0.85% saline whereupon cells were plated on M9-glucose plates supplemented with leucine, thiamine and streptomycin sulfate (150 µg mL^-1^) for measuring total exconjugants and the above plus kanamycin (50 µg mL^-1^) for measuring transposon hops. Transposition frequencies were calculated by dividing the number of Kan^R^Sm^R ^colonies by the number of Sm^R ^colonies.

### EDC/NHS chemical cross-linking

Transpososome assembly reactions (100X volume - 1 mL) were prepared with unlabeled ME DNA (500 nM), purified transposase (565 nM), and WT H-NS (1.2 µM) as previously described [9]. Reactions were concentrated by microfiltration from the initial volume to 0.045 mL (Millipore [Billerica, MA, USA] Vivaspin 30,000 kDa cut-off). Samples were then treated with 9.5 µL of the chemical cross-linker EDC (50 mM) plus NHS (12.5 mM) (both prepared in water) for 2.5 minutes at room temperature. Non-denaturing load dye was added to the cross-linking reactions and samples were applied to a 5% native polyacrylamide gel. After staining the gel with ethidium bromide the transpososomes and H-NS-bound transpososomes were isolated based on mobility differences. Proteins were eluted out of the gel slices at 42°C with 1 mL of elution buffer (0.5% SDS and 1 M sodium acetate), concentrated as described above and subjected to immunoblot analysis as previously described [22]. Purified Tn5 transposase and H-NS were also subjected to EDC/NHS cross-linking as above except that 0.5 µg (1.2 µM of transposase and 3.6 µM of H-NS) of each protein was used either separately or together with or without 2.5 µL of the chemical cross-linker EDC (50 mM) plus NHS (12.5 mM) and incubated at room temperature for three minutes. SDS load dye was added and samples were immediately loaded on the SDS protein gel for immunoblot analysis. For complexes that were treated with micrococcal nuclease, the cross-linking protocol was similar except for the following changes. After cross-linker treatment the reactions were stopped with addition of Tris-HCl pH 8.0 to a final concentration of 500 nM. Micrococcal nuclease (100 units) and 5 mM CaCl_2 _were added to the reactions and incubated at 37°C for 15 minutes. At the same time, additional samples were mock-treated with only 5 mM CaCl_2 _and no nuclease. A small amount of each sample (4 µL) was removed and analyzed on a 1% agarose gel to ensure that no DNA remained in the samples treated with the nuclease and that complexes were not disrupted in the mock-treated samples. The remainders of the samples were concentrated as described above to 20 µL and loaded on an SDS protein gel.

### MALDI-TOF MS analysis of cross-linked species

A 400x H-NS-transpososome assembly reaction was concentrated to 225 µL, divided into four equivalent aliquots and each aliquot was treated similarly to above. The cross-linking reactions were quenched by addition of Tris-HCl pH 8.0 to a final concentration of 500 nM and then applied to a 5% native polyacrylamide gel. H-NS-transpososome was eluted from the native gel after staining with ethidium bromide and fractions were pooled, concentrated and applied to a single lane of an SDS protein gel, which was stained with Coomassie Blue. Transposase monomer, H-NS monomer and cross-linked product 'a' were gel-isolated from the same lane using an Ettan Spot-picker (GE Healthcare - Mississauga, ON, Canada). In-gel digestion was performed using a MassPREP automated digester station (PerkinElmer). Gel pieces were Coomassie destained using 50 mM ammonium bicarbonate and 50% acetonitrile, which was followed by protein reduction using 10 mM dithiotreitol (DTT), alkylation using 55 mM iodoacetamide (IAA), and tryptic digestion. Peptides were extracted using a solution of 1% formic acid and 2% acetonitrile and lyophilized. Prior to mass spectrometry analysis, dried peptide samples were re-dissolved in 50% acetonitrile and 0.1% trifluoroacetic acid (TFA). A saturated solution of the MALDI matrix, α-cyano-4-hydroxycinnamic acid (CHCA), was prepared in 67% acetonitrile and 0.05% TFA, diluted to 70% saturation, mixed with the samples at 1:1 ratio (v/v) and 1 µL samples were spotted on the MALDI target. Mass spectrometry data were obtained using a 4700 Proteomics Analyzer, MALDI TOF (Applied Biosystems, Foster City, CA, USA). Data acquisition and data processing were done using MassLynx 3.5 Mass Spectrometry Software (Waters), respectively. The instrument is equipped with a 355 nm Nd:YAG laser; the laser rate is 200 Hz. Reflectron and linear positive ion modes were used. Reflectron mode was calibrated at 50 ppm mass tolerance. Each mass spectrum was collected as a sum of 1,000 shots. Theoretical masses of the peptides produced from a tryptic digestion of Tn5 transposase and H-NS were calculated using PROWL's ProteinInfo peptide mass prediction tool (Rockefeller University, Laboratory of Mass Spectrometry and Gaseous Ion Chemistry).

### Quantitative RT-PCR and analysis

A total of 1 to 2 mL of cells were removed from donor strain cultures immediately before the remaining 1 mL were mixed with recipient strain for the mating out. The cells were gently centrifuged (4000 × g for four minutes), resuspended in 200 µL of RNALater (Ambion - Burlington, ON, Canada and incubated at 4°C overnight. The following day, RNA was extracted from each sample using the RNeasy Mini-kit (Qiagen - Streetsville, ON, Canada). The quality and quantity of the final RNA samples were assessed using agarose gel electrophoresis and a NanoDrop spectrophotometer (IMPLEN) to measure A_260_, A_260/230 _and A_260/280 _ratios. A portion of the RNA was treated with the RNase-free TURBO DNA-free Kit (Applied Biosystems) as per instructions for typical amounts of contaminating genomic DNA. The resulting RNA was quantified again as above to ensure A_260/230 _and A_260/280 _ratios were in the range of 1.5 to 2.0. RT-PCR was performed with a portion of this RNA (0.5 to 1 µg) using the protocol and reagents in the High Capacity RT-PCR Kit (Applied Biosystems). The final cDNA concentrations of 25 to 50 ng µL^-1 ^(depending on the starting amount of RNA) were diluted to 25 ng µL^-1 ^(if necessary) for use in real-time PCR reactions. TaqMan primers and probes (see Table 1) were designed using the Applied Biosystem Primer Express 2.0 software to the very 5' end (nucleotides 5 to 87) of the transposase transcript and the endogenous control 16S rRNA. 16S RNA was used as a 'normalizing' control for Δ*hns *strains in other work [26]. Reactions for real-time PCR were done in 20 µL volumes in 384 well clear plates using the protocol in the TaqMan Gene Expression MasterMix guide (Applied Biosystems). Three biological replicates were tested per strain and three technical replicates were used for real-time PCR for each biological replicate. Appropriate control reactions were conducted where RNA was omitted, or reverse transcriptase was omitted for each RNA sample. Control real-time PCR reactions containing no cDNA (1X TE replacement) and no TaqMan 'enzyme mix' were also conducted. Reactions were run using standard cycle parameters on an Applied Biosystems 7900-HT Real-Time System. The Pfaffl mathematical model of relative quantification was used to determine the relative amounts of transposase mRNA [40]. Relative amount = (*E*_transposase_)^ΔCt*transposase*^/(*E*_16S_)^ΔCt*16S *^where *E *is the PCR amplification efficiencies of the transposase transcript and 16S rRNA transcript that were determined by creating several standard curves with known diluted target concentrations (*E *= 10^(-1/slope) ^). The amplification efficiencies for the transposase target and 16S gene using these primers and probes were determined to be 1.99 and 1.92, respectively. ΔCt of transposase or 16S represents the average sample Ct for each condition subtracted from the average Ct of the reference condition to which all samples will be quantified relative to (that is, NK5830F' (*hns *+ strains) transformed with pDH533-4 (WT ME DNA)).

### Modeling H-NS-transpososome complex

The H-NS DNA-binding domain was manually positioned into the minor groove of the transposon end DNA, using the H-NS DNA-binding domain in complex with duplex DNA as a guide [23]. The symmetry of the Tn5 transpososome was used to place a second H-NS DNA-binding domain in an equivalent position on the second transposon end. For the 'front face' complex, the structure of the H-NS N-terminal domain (NTD) dimer [PDB:3NR7] was manually positioned to place the C-terminal ends close to the DNA-binding domains. Residues 83 to 90 were added to the C-terminal end of the H-NS NTD and the connection between the two domains was made using the loop-building utility in SwissPDBViewer [41]. A similar process was used to position the H-NS dimer on the opposite side ('back face') of the transpososome, but in this case H4 was manually positioned into the major groove of the transposon end DNA to facilitate the connection between the N- and C-terminal domains. Minor adjustments and corrections to the stereochemistry were made using Coot [42].

## Abbreviations

EDC: 1-ethyl-3-(3-dimethylaminopropyl)carbodiimide; EMSA: electrophoretic mobility shift assay; IE: inside end; ME: mosaic end; NHS: N-hydroxysuccinimide; RT-PCR: reverse transcriptase polymerase chain reaction; T'some: transpososome.

## Competing interests

The authors declare that they have no competing interests.

## Authors' contributions

CRW performed all of the experiments and helped to draft the manuscript. BS performed the structure-modeling studies and helped to draft the manuscript. DBH conceived of the study, participated in its design and coordination, and helped to draft the manuscript. All authors read and approved the final manuscript.

## Supplementary Material

Additional file 1 H-NS binding assays. Titration of H-NS into WT ME and OE transpososomes for K_d _calculations. Click here for file

Additional file 2 Mating out frequencies. Comparison of transposition frequencies in isogenic *hns *strains. Click here for file

Additional file 3 Plasmid copy number determination in mating out experiment. Agarose gel analysis of plasmid DNA preparations made from strains used in mating out experiments. Click here for file

Additional file 4 Relative levels of Tn5 transposase transcript in *hns *strains as measured by qRT-PCR. Standard curve used to determine the relative amounts of transposase mRNA. Click here for file
