# Supplementary material for: Identification of basepairs within Tn5 termini that are critical sfor H-NS binding to the transpososome and regulation of Tn5 transposition
Source: Mob DNA. 2012 Apr 13;3:7. doi: 10.1186/1759-8753-3-7 (PMC3347997; doi:10.1186/1759-8753-3-7)
Supplement: Additional file 1 — H-NS binding assays. Titration of H-NS into WT ME and OE transpososomes for Kd calculations. [file 1759-8753-3-7-S1.PDF]

Whitfield CR, Shilton BS, and Haniford DB (2012) Identification of basepairs within Tn5 termini that are critical for H-NS binding to the transpososome and regulation of Tn5 transposition

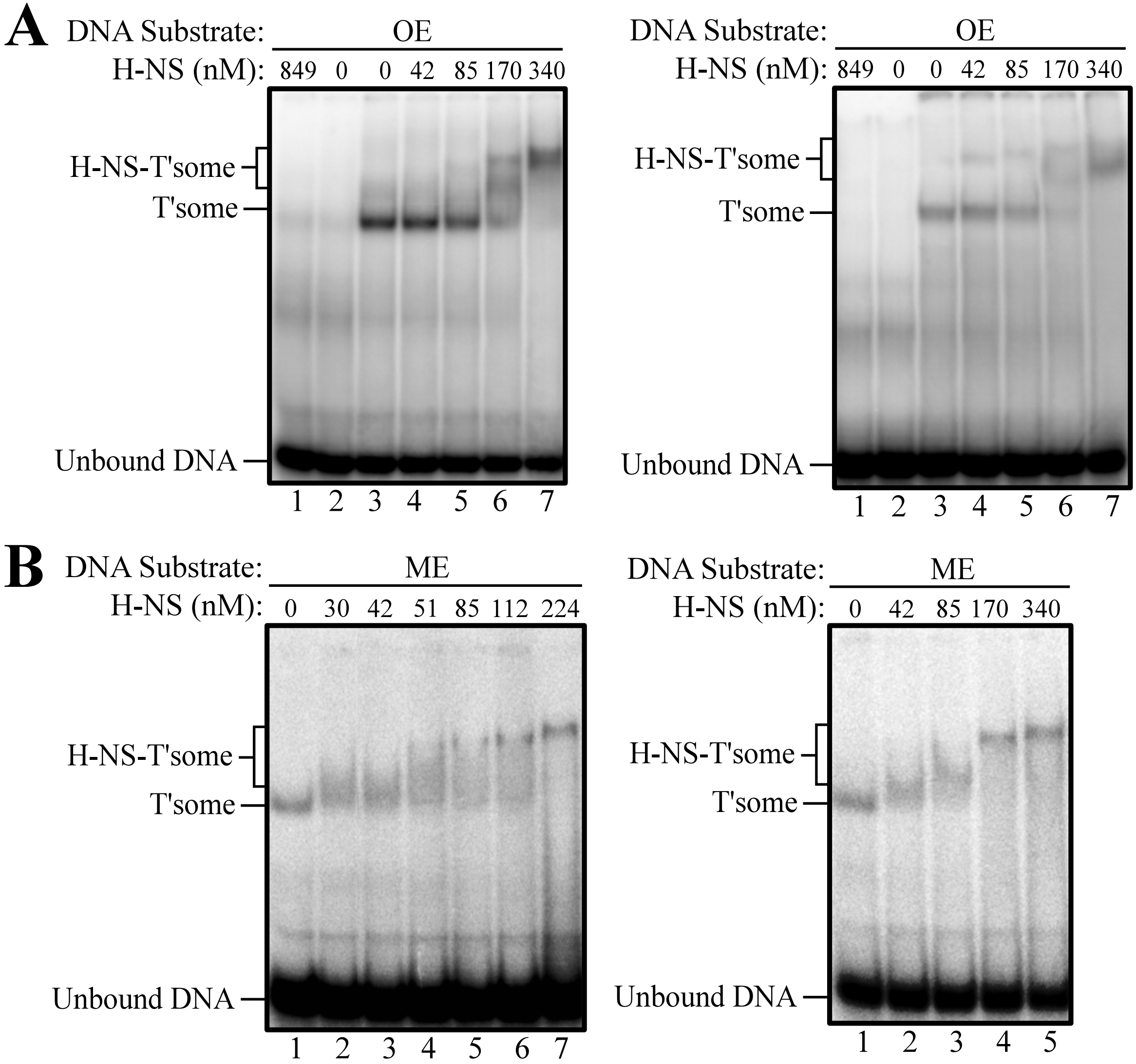

Additional File 1. H-NS binding assays. H-NS and transposase were added simultaneously to (A) OE and (B) ME ^{32}P-labelled transposon end DNA. In each experiment transposase and substrate DNA concentrations were kept constant, while the concentration of H-NS was varied as indicated. For a detailed explanation of the reaction assembly see the 'Methods' section within the article. Binding reactions were analyzed by gel electrophoresis on native 5% polyacrylamide gels and subject to phosphorimaging. Along with the results presented in Figure 2, data were fit to a quadratic equation as described in the 'Methods' section. The fits were used to provide estimates for the observed dissociation constant, obs K_d. Positions of transpososome (T'some), H-NS-bound transpososome (H-NS-T'some) and unbound substrate DNA are indicated.
