# Supplementary material for: Identification of basepairs within Tn5 termini that are critical sfor H-NS binding to the transpososome and regulation of Tn5 transposition
Source: Mob DNA. 2012 Apr 13;3:7. doi: 10.1186/1759-8753-3-7 (PMC3347997; doi:10.1186/1759-8753-3-7)
Supplement: Additional file 2 — Mating out frequencies. Comparison of transposition frequencies in isogenic hns strains. [file 1759-8753-3-7-S2.PDF]

Whitfield CR, Shilton, BS and Haniford DB (2012) Identification of basepairs within Tn5 termini that are critical for H-NS binding to the transpososome and regulation of Tn5 transposition

Additional file 2.

Table 1. *In vivo* transposition of wild-type and mutant mini-Tn5-Kan^R elements in isogenic *hns*+ and Δ*hns* strains with transposase supplied in trans (pDH641).

Transposon Source	Strain	Transposition frequency[a]	Normalized frequency[b]	Normalized frequency/pair[c]
pDH626(WT ME)	NK5830F'wild-type	2.1 (±0.9) x 10^{-4}	1.0	1.0
pDH626	NK5830F'Δ*hns*	3.7 (±2.1) x 10^{-5}	0.18	0.18***
pDH689(OE)	wild-type	1.2 (±0.7) x 10^{-3}	5.7	1.0
pDH689	Δ*hns*	3.2 (±1.2) x 10^{-4}	1.5	0.26**
pDH660(ME 8/9)	wild-type	4.5 (±2.7) x 10^{-6}	0.021	1.0
pDH660	Δ*hns*	2.1 (±1.0) x 10^{-6}	0.010	0.48*
pDH685(ME 3)	wild-type	1.2 (±0.6) x 10^{-5}	0.057	1.0
pDH685	Δ*hns*	1.3 (±0.8) x 10^{-5}	0.062	1.1

a. Relative transposition frequencies were calculated by dividing the number of Sm^{R}Kan^R colonies (transposition events) by the number of Sm^R colonies (total exconjugants) obtained per 0.1 mL of mating mix. Transposition frequencies represent an average value obtained from three independent experiments wherein matings with 3-5 different donor transformants were carried out.

b. Transposition frequencies were normalized to the level of transposition in the WT strain transformed with pDH626.

c. Each pair of transposition frequencies for each transposon was normalized to the WT strain for that specific pair. Asterisks indicate statistical significance for normalized transposition frequencies for each transposon pair as determined using the t-test. *$p<0.05$, **$p<0.005$, ***$p<0.0001$
