# Supplementary material for: Identification of basepairs within Tn5 termini that are critical sfor H-NS binding to the transpososome and regulation of Tn5 transposition
Source: Mob DNA. 2012 Apr 13;3:7. doi: 10.1186/1759-8753-3-7 (PMC3347997; doi:10.1186/1759-8753-3-7)
Supplement: Additional file 4 — Relative levels of Tn5 transposase transcript in hns strains as measured by qRT-PCR. Standard curve used to determine the relative amounts of transposase mRNA. [file 1759-8753-3-7-S4.PDF]

Whitfield CR, Shilton BS, and Haniford DB: Identification of basepairs with Tn5 termini that are critical for H-NS binding to the transpososome and regulation of Tn5 transposition

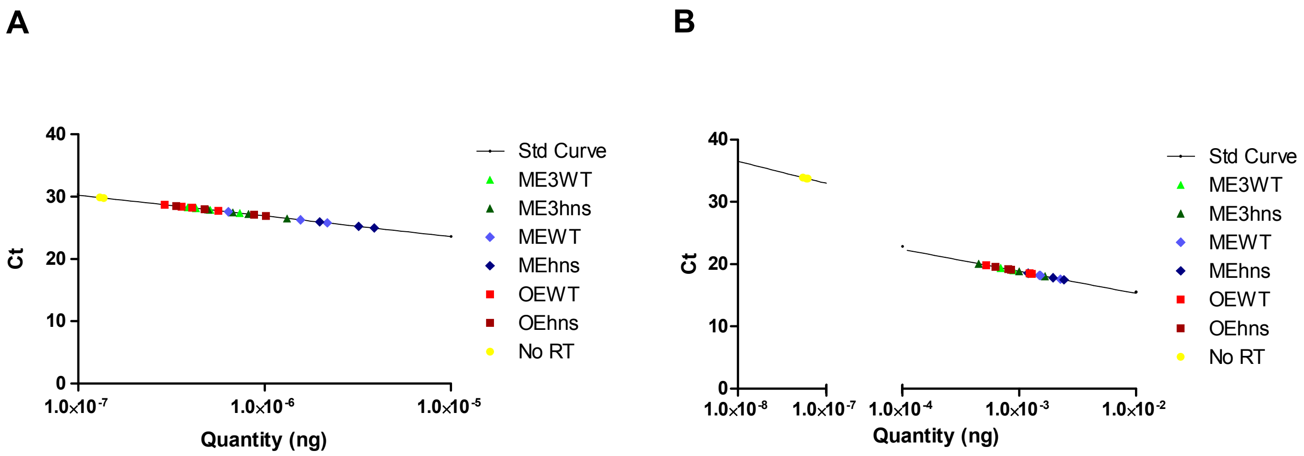

Additional file 4. Relative levels of Tn5 transposase transcript in hns strains as measured by qRT-PCR. For each condition, 3-4 independent cultures (each with 2-3 replicates), were used for qRT-PCR to determine the quantity of IS50 transposase (A) or 16S (B) transcript. A standard curve was used to determine the quantity of transcript based on the number of cycles required to reach a set threshold (Ct). Points on the graph represent quantity of transcript based on the mean Ct value of the replicates for each independent culture of the 6 conditions along with No RT controls.
