# Supplementary material for: Identification of basepairs within Tn5 termini that are critical sfor H-NS binding to the transpososome and regulation of Tn5 transposition
Source: Mob DNA. 2012 Apr 13;3:7. doi: 10.1186/1759-8753-3-7 (PMC3347997; doi:10.1186/1759-8753-3-7)
Supplement: Additional file 3 — Plasmid copy number determination in mating out experiment. Agarose gel analysis of plasmid DNA preparations made from strains used in mating out experiments. [file 1759-8753-3-7-S3.PDF]

Whitfield CR, Shilton BS, and Haniford DB (2012) Identification of basepairs within Tn5 termini that are critical for H-NS binding to the transpososome and regulation of Tn5 transposition

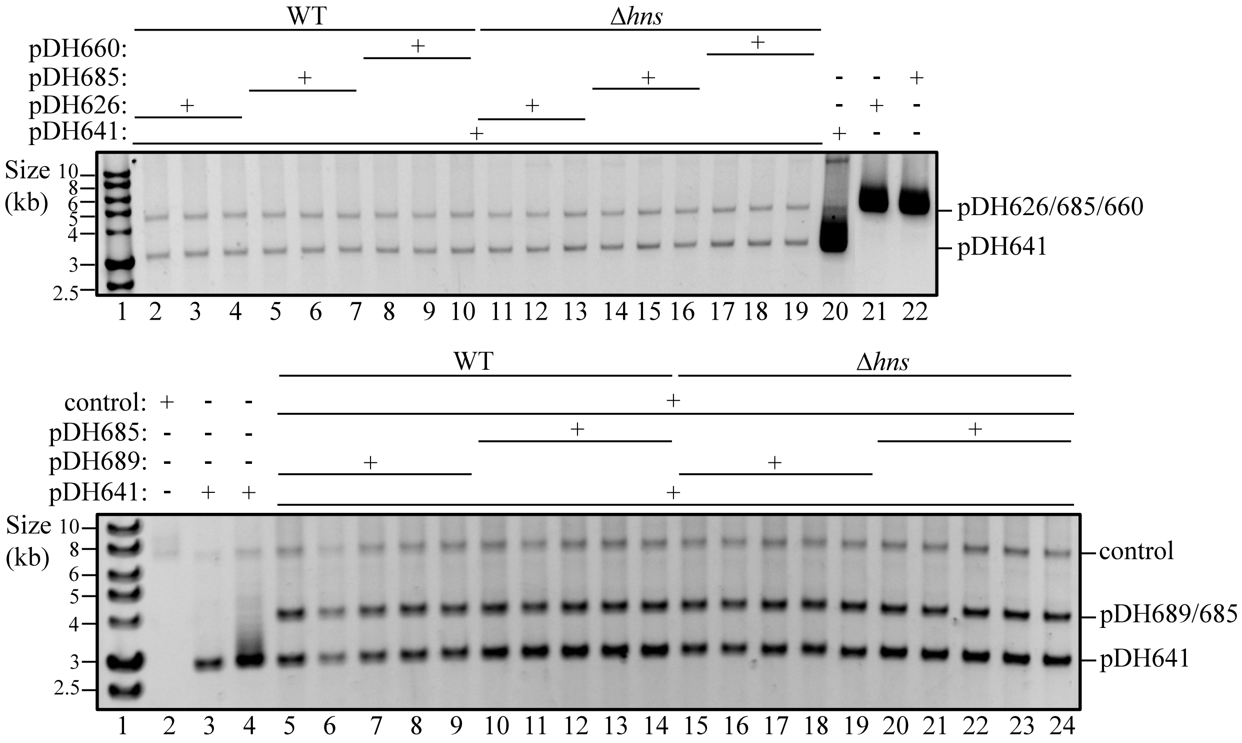

Additional file 3. Plasmid copy number determination in mating out experiment.
Copy number was determined immediately prior to mating to ensure that hns status did not influence the copy number or maintenance of any plasmid. Briefly, the optical density was determined for each sample to provide a rough estimate of the amount of cells. Equivalent cell density amounts from each sample were used for plasmid isolation using a Mini-prep kit (Sigma-Aldrich). In the bottom gel, an additional control was carried out to account for differences in plasmid recovery. An equivalent density of cells from DH5α transformed with a control plasmid of different size than the transposition plasmids was added to each donor sample before plasmid isolation was conducted. DNA from each sample (~5-10 μL) (along with plasmid control samples from previous plasmid isolations to indicate proper sizes) were run on a 1% agarose gel, stained with ethidium bromide, and viewed using an AlphaImager gel-doc system (AlphaInnotech).
